# Genital infections and reproductive complications associated with Trichomonas vaginalis, Neisseria gonorrhoeae, and Streptococcus agalactiae in women of Qom, central Iran

**Published:** 2017-06

**Authors:** Mahmoud Nateghi Rostami, Batool Hossein Rashidi, Azam Habibi, Razieh Nazari, Masoumeh Dolati

**Affiliations:** 1 *Department of Parasitology, Pasteur Institute of Iran, Tehran, Iran.*; 2 *Department of Microbiology and Immunology, Faculty of Medicine, Qom University of Medical Sciences, Qom, Iran.*; 3 *Department of Obstetrics and Gynecology, Vali-Asr Reproductive Health Research Center, Tehran University of Medical Sciences, Tehran, Iran.*; 4 *Department of Microbiology, Science and Research Branch, Islamic Azad University, Arak, Iran.*; 5 *Department of Microbiology, Islamic Azad University, Qom Branch, Qom, Iran.*; 6 *Cellular and Molecular Research Center, Qom University of Medical Sciences, Qom, Iran.*

**Keywords:** Sexually Transmitted Infections, Trichomoniasis, Gonorrhea, Pregnancy-related complications

## Abstract

**Background:**

*Trichomonas vaginalis *(*T.vaginalis*) and *Neisseria gonorrhoeae* (*N.gonorrhoeae*) are two most common non-viral sexually transmitted infections in the world. No data are available regarding the epidemiology of genital infections in women of Qom, central Iran.

**Objective:**

Epidemiological investigation of sexually transmitted infections in genital specimens of women referred to the referral gynecology hospital in Qom, central Iran.

**Materials and Methods:**

Genital swab specimens were collected from women volunteers and used for identification of bacterial and protozoal infections by conventional microbial diagnostics, *porA *pseudo gene LightCycler^®^ real-time PCR (for *N.gonorrhoeae*) and ITS-PCR (for *T.vaginalis*).

**Results:**

Of 420 volunteers, 277 (65.9%) had genital signs/symptoms, including 38.3% malodorous discharge, 37.9% dyspareunia, and 54.8% abdominal pain. Totally, 2 isolates of *Streptococcus agalactiae *were identified. Five specimens (1.2%) in Thayer-Martin culture and 17 (4.1%) in real-time PCR were identified as *N.gonorrhoeae*. Fifty-four specimens (12.9%) in wet mount, 64 (15.2%) in Dorset’s culture, and 81 (19.3%) in ITS-PCR showed positive results for *T.vaginalis*. Five mixed infections of *T.vaginalis*+ *N.gonorrhoeae *were found. The risk of *T.vaginalis *infection was increased in women with low-birth-weight (p*=*0.00; OR=43.29), history of abortion (p*=*0.00; OR=91.84), and premature rupture of membranes (PROM) (p*=*0.00; OR=21.75). The probability of finding nuclear leukocytes (p*=*0.00; OR=43.34) in vaginal smear was higher in *T.vaginalis *infection.

**Conclusion:**

The significant prevalence of trichomoniasis and gonorrhea emphasizes the need for accurate diagnosis and effective surveillance to prevent serious reproductive complications in women.

## Introduction

As estimated by the World Health Organization, 350-500 million cases are annually identified as harboring *Treponema pallidum*, *Neisseria gonorrhoeae (N.gonorrhoeae), Chlamydia trachomatis (C.trachomatis) *and *Trichomonas vaginalis (T.vaginalis) *which all classified as sexually transmitted infections (STIs) (1). More than 1 million people are infected by an STI every day, with the largest proportion in the region of south and south-east Asia. Totally, over 30 bacterial, viral and parasitic pathogens are causative agents of STIs (2). The last published report of World Health Organization estimated that globally 3.0 million adults are infected with *C.trachomatis*, 1.0 million with *N.gonorrhoeae*, and 13.2 million with *T.vaginalis *(3). 

*T.vaginalis* is incriminated as the leading agent of non-viral STI around the world. Besides, trichomoniasis is a neglected parasitic infection demanding more attention to be implemented by health authorities (4). Trichomoniasis is frequently found in women of reproductive age and also in women suffering from pregnancy-related complications (5-7). *N.gonorrhoeae *is a bacterial agent engaged in STIs which nowadays attracted more attention due to the emergence of resistant strains especially to extended-spectrum cephalosporins as the antibiotic of choice. In addition, the estimated incidence of new cases of gonorrhea is being increased recently (8).

Another bacterial species, *Group B streptococci* (GBS) or *Streptococcus agalactiae*, is also involved in genital infections. Approximately 10-30% of pregnant women are being recognized to be colonized with GBS, particularly in vagina or rectum (9). GBS infection affects both the mother and neonate, however, the serious complications are predominantly observed in neonates with GBS (10). Around half of infants born to untreated GBS-positive women become colonized and 1-2% of them develop the invasive disease (8). The most common clinical syndromes of early onset disease are sepsis and pneumonia which may present within the first 24-48 hr of life (9). Late-onset GBS infection is acquired by infants after 1 week of age and in up to half of cases manifests with meningitis (12).

STIs are not only the infections enduring the women of reproductive age but also coupled to adverse complications such as abortion, stillbirth, ectopic pregnancy, increased possibility of human immunodeficiency virus transmission, and pelvic inflammatory disease as the main cause of infertility (5, 10, 11). However, the majority of women with genital infections are asymptomatic and early diagnosis of STIs is necessary in order to give appropriate treatment to prevent serious complications. As a holy city, Qom is a major destination for millions of pilgrims every year. Moreover, Qom is a cosmopolitan city that houses thousands of seminarians and immigrants from different geographical regions around the world. This huge number of travelers and immigrants make Qom an important province in regard of epidemiologic investigations of STIs. 

In this study, we report the prevalence of *T.vaginalis*, *N.gonorrhoeae* and GBS infections in genital swab specimens collected from women of Qom, Iran. This information could be employed in the improving management of STIs to prevent reproductive consequences, and in the education of high-risk populations.

## Materials and methods

**Population and sampling**

From May 2013 to August 2014, all women aged 18-50 yr who admitted to Referral Gynecology Hospital from more than 15 health clinics of Qom city because of genital complaints or for regular examination were recruited. Exclusion criteria included administration of systemic or topical antibiotics within one month prior to sampling. After examination of each volunteer by a gynecologist, demographic data, history and clinical signs/symptoms were recorded in a questionnaire. Five endocervical/vaginal swab samples were collected from each volunteer under aseptic conditions and used for smear preparation, culture, and DNA extraction. 

**Examination of cellular morphology**

In addition to bacterial and protozoal diagnosis, the morphology of shedding cells was examined in vaginal discharge under the microscope. Vaginal smears were stained with Giemsa (Sigma, St. Louis, MO, USA) after fixation by methanol (Merck, Darmstadt, Germany) and examined for the characteristics of epithelial cells, polymorphonuclear leukocytes (PMN), and other cells by light microscopy (Carl Zeiss, Germany).

**Detection of** ***N.gonorrhoeae*** **infection**

For *N.gonorrhoeae* isolation modified Thayer-Martin agar (mTM) medium was prepared from Blood Agar Base No.2 (Oxoid, UK) supplemented with 5% sheep blood and Vancomycin, Colistin, Nystatin, and Trimethoprim (VCNT) antibiotics (Sigma, St. Louis, MO, USA). Swabs were cultured on modified TM agar and plates were kept on candle jar and incubated at 37^o^C for 48 hr. Preliminary identification of *N.gonorrhoeae *was done based on colony characteristics, carbohydrate utilization and biochemical reactions including catalase, oxidase, and superoxol tests. 

DNA extraction was carried out on the vaginal/endocervical samples by using AccuPrep^®^ Genomic DNA Extraction Kit according to manufacturer’s instruction (Bioneer, South Korea). A real-time PCR assay targeting the *porA *pseudogene was performed as described by Whiley (12). Briefly, LightCycler^®^ 8-Tube strips were used, each well of 20 µL reaction mixture contained: 10 µl of 2x FastStart Essential DNA Green Master (Roche Diagnostics GmbH, Germany), 2 µl of primer pairs mix (0.5 µM each) NIS.F 5'-CGGTTTCCGTGCGTTACGA-3′, and NIS.R 5'-CTGGTTTCATCTGATTACTTTCCA-3′, 5 µL of DNA extract, and up to 20 µL sterile PCR-grade water. 

PCR amplification was performed on the LightCycler^®^ system (Roche Diagnostics GmbH, Germany) with the following program: initial denaturation at 95^o^C for 10 min followed by 45 cycles of denaturation at 95^o^C for 10 sec, annealing at 55^o^C for 10 sec, and extension at 72^o^C for 20 sec. For normalizing the differences in the amount of input DNAs, the housekeeping gene 16s ribosomal RNA gene was selected as the internal standard and primer pairs Int. F 5′-AAC TGG AGGAAGGTGGGGA-3′ and Int. R 5′-AGG AGGTGATCCAACCGCA-3′ were used to amplify a 340 bp conserved region. *N.gonorrhoeae *ATCC 49226 was used as a standard reference strain. 

**Detection of recovered bacteria**

For isolation of Gram negative and Gram positive bacteria, the swab samples from genital discharge were plated onto Chocolate agar and incubated at 37^o^C for 24-48 hr. Grown colonies after Gram staining were subcultured on McConkey agar (for Gram-negative) or Trypticase soy agar (for Gram-positive). A combination of biochemical tests including Triple Sugar Iron, Methyl Red-Voges Proskauer, Sulfide Indole Motility, Urease, Simmons Citrate, and Lysine decarboxylase was used for identification of Gram-negative bacteria (All media from Merck, Darmstadt, Germany). API^®^ 20E kit (bioMérieux, France) was used for confirmation. A combination of biochemical tests including Catalase, Coagulase, Mannitol Salt Agar, Novobiocin and Bacitracin disk sensitivity, growth at 6.5% NaCl broth, Bile Esculin, Hippurate hydrolysis and CAMP test was used for final identification of Gram-positive bacteria including *S.agalactiae *(=GBS).

**Detection of** ***T.vaginalis*** **infection**

Wet mount smears were prepared in saline and examined immediately after sampling for identification of *T.vaginalis *under the light and phase contrast microscope (Carl Zeiss, Germany). Under the microscope, *T.vaginalis *was diagnosed based on morphological characteristics of trophozoites forms. Trophozoites are flagellated oval cells with jerky non-directional motility and a size of about 10x15 µm on average. Biphasic Dorset’s culture medium prepared from eggs for solid phase overlaid with Ringer solution containing rice starch for liquid phase (13, 14). Swabs were inoculated into the liquid phase and media were kept at 37^o^C for 7 days. The media were checked every day and positive samples were approved after viewing motion of live parasites under the microscope. 

For molecular detection of *T. vaginalis*, genomic DNA was extracted using the conventional phenol-chloroform procedure. Briefly, to 100 μl of each specimen, 200 μl of lysis buffer (100 mM Tris-HCl, pH 8; 10 mM ethylene-diamine tetra acetic acid (EDTA), pH 8; 1% sodium dodecyl sulfate; 100 mM NaCl; 2% Tween 20) (Sigma, St. Louis, MO, USA) with 30 μl proteinase K (100 mg/ml) was added and incubated at 65^o^C for 1 hr. After centrifugation, the supernatant was transferred to a new microtube and the equal volume of phenol-chloroform-isoamyl alcohol (25:24:1) was added, vortexed, and centrifuged at 10000 rpm, for 10 min. The supernatant was transferred to a new microtube and chloroform extraction was performed again. Then twice the volume of absolute ethanol and 1/10 volume of 3 M sodium acetate (pH=5.2) were added to the supernatant and incubated at -20^o^C for 1 hr and centrifuged at 12000 rpm for 10 min.

The precipitant was then washed with 70% ethanol by centrifugation at 12000 rpm for 5 min. The pellet was air-dried and resuspended in 30 μl of distilled water and stored at -20^o^C until use. Primer pairs TV.F 5'-CGG TAG GTG AACCTGCCGTTGG-3' and TV. R 5'-AGT TCAGCGGGTCTTCCTGCG-3' which amplify the internal transcribed spacer (ITS) ribosomal RNA region were used (15). 

Amplification was carried out in a total volume of 25 µl containing 2.5 µl 10x PCR buffer, 1.5 mM MgCl_2_, 0.5 µl of each (25 pmol/ml), 0.2 mM of each dNTPs, and 0.5 U *Taq* DNA polymerase (Fermentas Life Sciences, York, UK). The conditions for *T.Vaginalis *conventional PCR were as follows: initial denaturation of 94^o^C for 5 min, then 35 cycles of 94^o^C for 30 sec, 61^o^C for 30 sec, 72^o^C for 45 sec, followed by final extension of 72^o^C for 10 min. (Mastercycler^®^, Eppendorf, Germany). For confirmation of the results, some *T.vaginalis *PCR products were submitted for sequencing.

**Ethical consideration**

The proposal of this study was approved by Ethical Committee of Qom University of Medical Sciences (MUQS) with the reference number P-34/18958. All volunteers agreed to participate in the investigation and signed a written informed consent. 

**Statistical analysis**

Data processing and the statistical analysis were performed using Statistical Package for the Social Sciences, version 18.0 (SPSS Inc, Chicago, Illinois, USA) and GraphPad Prism v.5.01 (GraphPad Software Inc., La Jolla, CA, USA) softwares by Pearson chi-square test, Fisher’s exact test, and univariate binary logistic regression.

## Results

Totally, 420 women volunteers were enrolled in this study with the mean±SD age of 33.74±8.21 years. Among them, 399 (95%) were Iranian and 21 (5%) were foreign immigrants. All the women were married and 17 were divorced (4.1%). All women had a unique sexual partner, 7 (1.7%) were pregnant, and most of the women (27.5%) had 2 kids at the time of sampling (range 0-7). Based on data, 309 (73.6%) women had using at least one of the contraceptive methods, including natural contraception as the most frequent (33.4%) method.

Among 420 volunteers, 277 (65.9%) had genital clinical signs/symptoms. Totally, 161 (38.3%) volunteers had malodorous discharge, 155 (36.9%) had itching, 91 (21.7%) had dysuria, 159 (37.9%) had dyspareunia, and 230 (54.8%) had low abdominal pain. Common pregnancy-related complications included 21 (5%) cases of low birth weight, 33 (7.9%) of infertility, 51 (12.1%) of premature rupture of membranes (1), 106 (25.2%) of history of abortion and 60 (14.3%) of curettage. On examination of Gram stained smears by microscopy, 47 (8.7%) of the specimens had no bacteria and 163 specimens (32.7%) had a combination of bacteria. 

Among the Gram positive cocci 9 isolates of *Enterococcus* spp., 4 isolates of *Staphylococcus saprophyticus*, 9 isolates of *S. aureus*, and 2 isolates of GBS were recovered. None of the pregnant women were infected with GBS. In Gram negative bacilli, 10 isolates of *E.coli* were identified. Of 420 specimens, *N.gonorrhoeae *infection was detected in 5 (1.2%) specimens using culture and biochemical tests. In real-time PCR assay, 17 (4.1%) specimens provided positive results for NG infection. Pearson Chi-square analysis showed no significant difference between the women with *N.gonorrhoeae *and those without *N.gonorrhoeae *infection with regard to the manifestation of clinical signs/symptoms. None of the pregnant women were infected with *N.gonorrhoeae*. 

**Figure 1:**
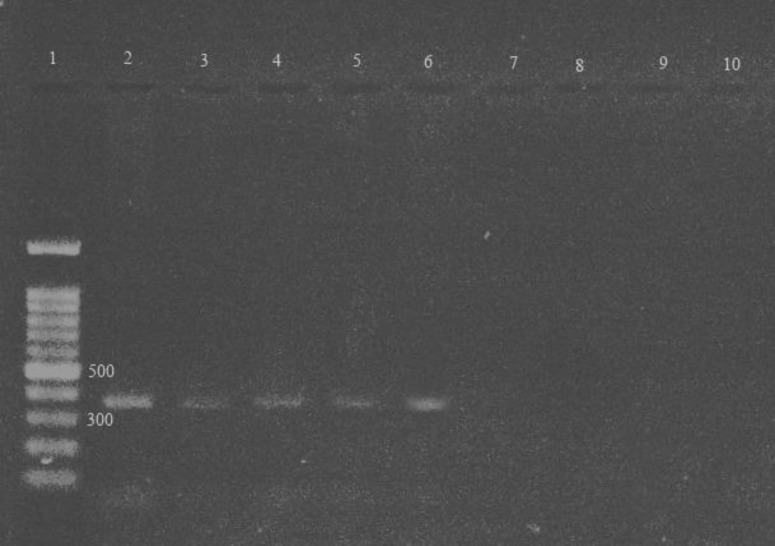
Results of PCR amplification of *T. vaginalis* DNA. Vaginal swab specimens were used for DNA extraction followed by ITS region PCR. A 361 bp amplicon is the representative of *T. vaginalis.*

In detection of *T.vaginalis *with wet mount examination, totally 12.9% of specimens (54/420) showed positive results and as shown in table I the rate of infection was significantly different between symptomatic (42/277) vs. asymptomatic (12/143) women (p=0.03). In Dorset’s culture, a total of 15.2% of specimens (64/420) showed positive results. In PCR test targeted to ITS region of *T.vaginalis*, a 361 bp band was observed after amplification (Figure 1) and totally 19.3% of specimens (81/420) showed positive results (Table I). None of the pregnant women were infected with *T.vaginalis*. Sequencing results showed that ITS ribosomal RNA region of *T.vaginalis *was correctly amplified via PCR. Sequence data were submitted to GenBank under the accession number KP221674.1.

Five mixed infections were identified including 3 specimens of *T.vaginalis *(by real-time PCR) + *N.gonorrhoeae *(by PCR), 1 specimen of *T.vaginalis *(by culture) + *N.gonorrhoeae *(by real-time PCR), 1 specimen of *T.vaginalis *(by PCR) + *N.gonorrhoeae *(by biochemical tests and culture). Women volunteers were categorized according to *T.vaginalis *infection test results. Pearson Chi-square analysis showed that manifestation of dysuria (p=0.00), low abdominal pain (p=0.03), itching (p=0.04), history of abortion (p=0.00), ectopic pregnancy (p=0.03), and low birth weight (p=0.02) were more common in *T.vaginalis *infected than *T.vaginalis *non-infected women. After exclusion of women with any pathogenic bacterial infection, totally 400 volunteers were regarded in the analysis of univariate binary logistic regression (Table II). 

As seen, analysis showed that the risk of having low birth weight was increased about 43-fold (p*=*0.00; OR 43.29; 95%CI 2.79-671.98), the risk of PROM about 21-fold (p=0.00; OR 21.75; 95%CI 4.12-136.95) and the risk of abortion about 91-fold (p*=*0.00; OR 91.84; 95%CI 15.51-544.23) in women with *T.vaginalis *compared to those without *T.vaginalis *infection. Also, the likelihood of finding PMN was increased about 43-fold (p*=*0.00; OR 43.34; 95%CI 2.82-665.17) in women with *T.vaginalis *compared to those without *T.vaginalis *infection (Table II).

**Table I.** Performance of different diagnostic tests in detection of genital infections in women grouped by symptom presentation

**Pathogens**		**Total (n=420)**	**Symptomatic women (n=277)**	**Asymptomatic** **women (n=143)**	**p-value** *
*Trichomonas vaginalis*					
	Wet mount	54 (12.9%)	42 (15.1%)	12 (8.4%)	0.03
	Culture	64 (15.2%)	48 (17.0%)	16 (11.2%)	0.09
	ITS-PCR	81 (19.3%)	57 (20.6%)	24 (16.8% )	0.12
*Neisseria gonorrhoeae*					
	Biochemical and Culture	5 (1.2%)	2 (0.7%)	3 (2.1%)	0.72
	Real-time PCR	17 (4.05%)	9 (3.2%)	8 (5.6%)	0.45

All data presented as absolute frequency and percentages.

ITS-PCR= Internal transcribed spacer-Polymerase chain reaction

* Pearson Chi-square analysis

**Table II:** Results of regression analysis in two groups of women categorized based on genital trichomoniasis infection

**Characteristics**		**Non-Trichomoniasis group**	**Trichomoniasis group**	**p-value**	**OR**	**95% CI**
Signs/Symptoms						
	No *	92 (28.7%)	31 (38.1%)	1.00	0.00	0.00-0.02
	Yes	227 (71.3%)	50 (61.9%)			
Nationality						
	Iranian *	304 (95.3%)	75 (92.6%)	0.99	1.22	0.35-6.27
	Immigrants	15 (4.7%)	6 (7.4%)			
Contraception						
	No *	77 (24.1%)	20 (24.6%)	0.63	1.47	0.30-7.18
	Yes	242 (75.9%)	61 (75.4%)			
Cellular morphology						
	No PMN *	270 (84.7%)	8 (10.2%)	0.00	43.34	2.82-665.17
	PMN	49 (15.3%)	8 (10.2%)			
Antibiotic administration						
	No *	261 (81.9%)	73 (9.7%)	0.08	5.63	0.30-22.97
	Yes	58 (18.1%)	8 (9.3%)			
History of chronic diseases						
	No *	273 (85.5%)	74 (91.5%)	0.09	2.95	0.90-36.75
	Yes	46 (14.5%)	7 (8.5%)			
Dysuria						
	No *	236 (74.1%)	73 (90.0%)	--------	-------	---------
	Yes	83 (25.9%)	8 (10.0%)	0.99	0.00	0.00
Malodorous discharge						
	No *	188 (58.9%)	52 (64.4%)	--------	-------	---------
	Yes	131 (41.1%)	29 (35.6%)	0.05	6.30	0.96-41.28
Cervicitis						
	No *	309 (96.8%)	81 (100%)	--------	-------	-----------
	Yes	10 (3.2%)	0 (00.0%)	0.99	0.00	0.00
Low abdominal pain						
	No *	115 (36.2%)	48 (59.3%)	--------	-------	-----------
	Yes	204 (63.8%)	33 (40.7%)	0.31	0.42	0.71-2.26
Dyspareunia						
	No *	195 (61.0%)	60 (73.7%)	--------	-------	------------
	Yes	124 (39.0%)	21 (26.3%)	0.69	1.46	0.24-9.55
Itching						
	No *	189 (59.2%)	61 (75.4%)	--------	-------	------------
	Yes	130 (40.8%)	20 (24.6%)	0.88	1.22	0.26-9.73
Abortion						
	No *	260 (81.6%)	45 (55.1%)	--------	-------	------------
	Yes	59 (18.4%)	36 (44.9%)	0.00	91.84	15.51-544.23
Ectopic pregnancy						
	No *	318 (99.6%)	76 (94.1%)	--------	-------	------------
	Yes	1 (0.4%)	5 (5.9%)	0.99	0.00	0.00
Stillbirth						
	No *	311 (97.5%)	78 (96.6%)	--------	-------	------------
	Yes	8 (2.5%)	3 (3.4%)	0.36	0.11	0.01-12.53
Infertility						
	No *	293 (91.8%)	79 (97.5%)	--------	-------	------------
	Yes	26 (8.2%)	2 (2.5%)	0.99	0.00	0.00
Low birth weight						
	No *	311 (97.5%)	73 (90.7%)	--------	-------	------------
	Yes	8 (2.5%)	8 (9.3%)	0.00	43.29	2.79-671.98
PROM						
	No *	288 (90.4%)	70 (86.4%)	--------	-------	-----------
	Yes	31 (9.6%)	11 (13.6%)	0.00	21.75	4.12-136.95
Curettage						
	No *	169 (84.4%)	70 (86.4%)	--------	-------	------------
	Yes	50 (15.6%)	11 (13.6%)	0.01	0.03	0.01-0.37
Total		319 (100%)	81 (100%)			

OR= Odds ratio

CI= Confidence intervals

* Regarded as reference group

**Table III:** Summarized data on the prevalence rates of *T. vaginalis* infection in different populations

**Region**	**Population**	**Sample size**	**Method**	**Prevalence rate (%)**	**Reference no.**
Kermanshah, Iran	General population	600	Wet mount	1.5	41
			Dorset’s culture	2.1	
Hamadan, Iran	Clinically trichomoniasis	683	Wet mount	2.2	42
			Diamond culture		
Kashan, Iran	General population	970	Wet mount	1.97	16
			TYM culture	2.3	
Hamadan, Iran	General population	750	Wet mount	1.7	17
			Dorset’s culture	2.1	
Semnan, Iran	Pregnant women	1223	Wet mount	5.5	43
Ahwaz, Iran	Women with genital complaints	100	Wet mount	14	21
			Xenostrip-Tv^®^		
Dohok, Iraq	Women with genital complaints	425	Wet mount	2.4	44
			H & E stained smear	3.5	
			Diamond culture	5.4	
Basra, Iraq	General population	352	Wet mount	13	45
Sivas, Turkey	Women with genital complaints	258	Wet mount	1.9	46
			CPLM culture	1.6	
Antakya, Turkey	Women with genital complaints	275	Wet mount	1.8	47
			Culture	2.2	
Islamabad, Pakistan	Women with genital complaints	100	Wet mount	4	37
Lahore, Pakistan	Women selling sex	730	InPouch TV^®^ culture	5.1	48
Jeddah, Saudi Arabia	General population	1767	Wet mount	0.7	49
Saudi Arabia	General population	167	Wet mount	9	50
			InPouch TV culture		

## Discussion

Data in this study was drawn from the population who referred to the referral gynecology hospital and extending the prevalence rates to general population should be extrapolated with caution. Results of a recent systematic review estimated the overall prevalence rate of 8% (Range: from 0.009%-38.8%) for *T.vaginalis *infection in general population of Iran (16). According to different reports, the prevalence rates of *T.vaginalis *infection in the region lies in the range of 1.5%-6.4% by using wet mount and 2% to 4.56% by using culture method (Table III). However, the prevalence rates were found to be higher in central provinces of the country such as the capital Tehran, that had the mean prevalence rate of 15.3% (17). 

Based on the current data, the prevalence rate of *T.vaginalis *infection in the population was similar to the high prevalence of central part of Iran. There are reports showing that in women with complaints of vaginal symptoms, the prevalence rates of *T.vaginalis *infection were higher, raising up to 14% (18). In the current study, symptomatic women had a rate of 15-17% of *T.vaginalis *infection on wet mount examination or culture which is not significantly higher than similar reports. In the neighboring countries, *T.vaginalis *infection rates varied based on the region and the population (Table III). On PCR analysis, the higher rate of *T.vaginalis *infection was seen in this study which is comparable to some other reports from Iran (21). 

This high prevalence rate might be due to the characteristics of the population such as a high number of symptomatic women (65%), the significantly low level of education (82%) and mixed population of immigrant/ local women. In addition, people living in Qom composed of strict traditional religious population and their sociocultural conditions seem to be contributed in high prevalence rate of STIs. As data show, none of the volunteers were single, since the parents usually do not permit for genital examination of the girls. Even married women are not comfortable for sampling and because infected patients fear of stigma associated with STIs, do not refer to clinics in due time, remain undiagnosed and untreated, and disseminate the infection to sexual partners. 

All studies summarized in table III, have the drawback of using non-molecular diagnostics with low sensitivity. The sensitivity of wet mount method is estimated to be about 60-70% which falls to as low as 35% within half an hour after specimen collection, and sensitivity of culture methods is estimated to be ranged from 65% up to 95% (19). Based on the present data, wet mount method has not enough sensitivity for detection of *T.vaginalis *in genital specimens, especially in asymptomatic women. In one investigation, the urine and vaginal specimens of women were rechecked for *T.vaginalis *detection by using PCR and results showed that out of 161 negative samples by direct smear and culture, 7 samples (4.3%) turned positive by PCR (20). Similar to our finding, several reports from Iran support the higher prevalence rates of *T.vaginalis *infection in women when molecular techniques have been used (15, 18). Nucleic acid amplification-based tests have generally been more sensitive than traditional tests in the diagnosis of *T.vaginalis *and *N.gonorrhoeae*, but their high cost and need of infrastructure has limited their widespread use in laboratories (21).

The present data showed that PROM, low birth weight, and abortion are serious outcomes strongly associated with *T.vaginalis *infection. Other possible infectious etiologies of pregnancy-related complications were ruled out including common viral and bacterial genital infections; although, these complications might be resulted from other genitourinary disorders which have not been evaluated. These results are in agreement with earlier studies which suggested an association between *T. vaginalis *infection and serious adverse consequences including infertility and low birth weight infants (6, 7). Previous studies demonstrated that *T.vaginalis* infection in pregnancy is significantly correlated with an elevated risk of PROM (5) which is supported by a case-control study among pregnant women of Uganda (22). Another study, a cohort conducted on 13,816 cases, emphasized on the significant likelihood of a low birth weight infant and preterm delivery in *T.vaginalis *-infected pregnant women (5, 7).

The probability of finding PMN in the stained smear of vaginal discharge was higher in women with trichomoniasis. Following colonization in vaginal canal, *T.vaginalis* trophozoites secrete various mediators leading to local host inflammatory response and infiltration of PMN leukocytes and lymphocytes in the endometrial tissue of lower genital tract (6, 23).

In real-time PCR assay, *N.gonorrhoeae *was identified in 17 (4.05%) endocervical specimens. In contrast to *T.vaginalis*, information on the prevalence of *N.gonorrhoeae *in Iranian women is scarce and real-time PCR has not yet been used for identification of *N.gonorrhoeae*. Nasirian and colleagues reported an estimated incidence rate of 2.44 (95% CI: 1.17-6.65) per 1000 women for *N.gono... *(16). *N.gonorrhoeae *was detected in 2.38% of 294 endocervical swabs collected from women referred to gynecology clinics in Kashan, central Iran (24). However, examination of urine specimens from 209 infertile women as cases and 170 pregnant women as controls by PCR method revealed negative results for *N.gonorrhoeae *detection (25). A possible explanation for this result may be the lack of adequate sensitivity of conventional PCR for detection of *N.gonorrhoeae *in urine samples (21).

Before the development of nucleic acid amplification tests (NAATs), laboratory diagnosis of gonorrhea was limited to traditional methods including Gram stain and culture. In diagnosis of symptomatic gonorrhea in men, the Gram stain is a cost-effective and rapid method with comparable sensitivity to bacterial culture. However, the sensitivity and specificity are low for diagnosis of gonorrhea in women, especially when used for extragenital sites. Bacterial culture has a relatively good sensitivity and specificity for detection of gonorrhea infection from different specimens, the sensitivity is ranging from as low as 50% for chronic infections to about 90% for acute infections. 

Although culture provides a viable organism for antibiotic susceptibility assay, but the specimens need to be transported under appropriate conditions to maintain organism viability. Several advantages could be assumed for NAATs over routine methods in the diagnosis of *N.gonorrhoeae*. The sensitivity/ specificity of NAATs is generally higher than traditional methods such as culture, and as reported by some systematic reviews, pooled sensitivity of commercially available NAATs is ranged from %95 to 100% for detection of gonococcal infections in women (29,30). Considering the high efficiency, NAATs are appropriate tools to the early and accurate diagnosis of both symptomatic and asymptomatic women with *N.gonorrhoeae* infections.

In contrast to bacterial culture, NAATs do not require the viable organism, so the specimen collection and transportation is more practical for people of distant area. In addition, different types of NAATs with good performance are available which use noninvasive specimens such as urine samples for detection, this is more acceptable for women specially those who have religious and cultural limitations for genital specimen collection (25). In this study, 5 coinfections of *T.vaginalis* and *N.gonorrhoeae *were detected in genital specimens. In STI infected women, a significant portion is coinfected with two or more pathogens simultaneously. There are evidence suggesting that *T.vaginalis *infection of genital tract might be a clue for other serious STIs such as *N.gonorrhoeae*, *C.trachomtis*, as well as human immuno deficiency virus and human papillomavirus (26). 

Another important finding of this study is 2 cases of GBS. None of the GBS positive patients were pregnant. This rate of infection is lower than other studies in Iran that reported the prevalence of 9.1% in Shiraz (south of Iran), 20.6% in the capital Tehran, and 9.5% in Bushehr (south of Iran) (27). Also, various publications have reported the prevalence of GBS in pregnant women in neighboring countries, showing a higher rate of infection than this study. Instances are 10.6% in Turkey, 16.4% in Kuwait and 5% in Pakistan (28). 

In this study, the women were not satisfied with giving rectal samples. This might influence the prevalence of GBS in the present study and the rate would have been raised if recto-vaginal swabs had been collected. According to CDC guidelines, sampling of both the vagina and rectum increases the yield of GBS and in part of infected women, GBS can only be found in rectal specimens (29). Reports from different regions of Iran show that the prevalence of GBS is higher if recto-vaginal specimens are examined (30, 31).

In bacterial detection, in some specimens, neither pathogenic bacteria nor normal flora was recovered. If the quantity of vaginal microbiota is scarce in the specimen, it might be not detected in stained smear even be isolated in culture. Besides, unnecessary medical involvement and inappropriate self-treatment may lead to temporary elimination of normal flora.

## Conclusion

In summary, the significant prevalence of trichomoniasis and gonorrhea emphasizes the need for surveillance and screening of *T.vaginalis* and *N.gonorrhoeae* infection in women of Qom province. The present study provides additional evidence with respect to pregnancy related sequels of *T.vaginalis* infection. Since STIs is not routinely screened in asymptomatic women and the infection may persist for several months in the genital tract, early examination and accurate diagnosis of STIs by point-of-care molecular techniques could prevent serious reproductive complications such as low birth weight and abortion especially in *T.vaginalis* infected women. 

Due to the relatively significant prevalence of *N.gonorrhoeae* infection in the population, it is recommended that sexually active young women be tested for gonorrhea in screening program in healthcare settings. Based on the present data, awareness should be raised in high-risk populations including middle-aged women with genital signs/symptoms to check for possible infection or co-infections of STIs including *N.gonorrhoeae* and *T.vaginalis*. Since apparently, the prevalence of GBS is low-estimated in the current study, more comprehensive studies are needed to evaluate the true prevalence of GBS particularly in pregnant women. 

**Limitation**

This study was limited to detection of bacterial and protozoal infection on examination of vaginal swab specimens from women. Study of other STIs especially viruses such as human immunodeficiency virus and human papillomavirus and other specimens such as urine samples among the same population is recommended.
